# Different *Plasmodium falciparum* clearance times in two Malian villages following artesunate monotherapy

**DOI:** 10.1016/j.ijid.2020.03.082

**Published:** 2020-06

**Authors:** Aminatou Kone, Sekou Sissoko, Bakary Fofana, Cheick O. Sangare, Demba Dembele, Aboubecrine Sedhigh Haidara, Nouhoum Diallo, Aoua Coulibaly, Aliou Traore, Sekou Toure, Kadidia Haidara, Kassim Sanogo, Issaka Sagara, Khalid B. Beshir, José P. Gil, Ogobara K. Doumbo, Abdoulaye A. Djimde

**Affiliations:** a Malaria Research and Training Center, Faculty of Pharmacy, University of Science, Techniques and Technology of Bamako, Mali; b Faculty of Infectious and Tropical Diseases, London School of Hygiene & Tropical Medicine (LSHTM), London, UK; c Malaria research Center, Department of Microbiology and Tumor Cell Biology, Karolinska Institutet, Stockholm, Sweden

**Keywords:** Malaria, Artesunate monotherapy, Parasite clearance, qPCR, *Plasmodium falciparum*

## Abstract

**Background:**

Artemisinin resistance described as increased parasite clearance time (PCT) is rare in Africa. More sensitive methods such as qPCR might better characterize the clearance phenotype in sub-Saharan Africa.

**Methods:**

PCT is explored in Mali using light microscopy and qPCR after artesunate for uncomplicated malaria. In two villages, patients were followed for 28 days. Blood smears and spots were collected respectively for microscopy and qPCR. Parasitemia slope half-life was calculated after microscopy. Patient residual parasitemia were measured by qPCR.

**Results:**

Uncorrected adequate clinical and parasitological responses (ACPR) observed in Faladje and Bougoula-Hameau were 78% and 92%, respectively (p = 0.01). This reached 100% for both after molecular correction.

Proportions of 24H microscopy positive patients in Faladje and Bougoula-Hameau were 97.2% and 72%, respectively (p < 0.0001).

Slope half-life was 2.8 h in Faladje vs 2H in Bougoula-Hameau (p < 0.001) and

Proportions of 72H patients with residual parasitemia were 68.5% and 40% in Faladje and Bougoula-Hameau, respectively (p = 0.003). The mean residual parasitemia was 2.9 in Faladje vs. 0.008 in Bougoula-Hameau (p = 0.002).

Although artesunate is efficacious in Mali, the longer parasite clearance time with submicroscopic parasitemia observed may represent early signs of developing *P. falciparum* resistance to artemisinins.

## Highlights

• High prevalence of residual parasitemia at day 3 post-artesunate monotherapy treatment using qPCR while no parasites were detected by microscopy at the same timepoint.
• A longer parasite clearance time observed in a Malian village.
• Artesunate treatment is still efficacious on *Plasmodium falciparum* in Mali.

## 1 Introduction

Malaria is still a devastating disease in endemic regions despite major efforts for its control and elimination. Artemisinin-based Combination Therapies (ACT) are first line treatment for malaria case management (WHO, 2015). Combining fast acting artemisinin- which drastically reduces the parasite biomass- with a long-lasting partner drug that clears the remaining parasitemia would protect both drugs from parasite resistance (Davis et al., 2005).

First decreased efficacy of Plasmodium falciparum to artemisinins observed *in vivo* was reported in South-east Asia and defined as a delay in clearance of parasite as measured by light microscopy (Noedl et al., 2008, Dondorp et al., 2009, WWARN, 2010, Flegg et al., 2013). Treatment failures were increasingly observed during clinical trials in the Greater Mekong regions (Dondorp et al., 2009). Resistance to artemisinin based combination therapies (ACTs) or to artemisinin derivatives in monotherapy were observed in several places in Asia (Cheeseman et al., 2012, Phyo et al., 2012, Ashley et al., 2014, Takala-Harrison et al., 2014, Imwong et al., 2017). The short half-life of artemisinins in patients (Navaratnam et al., 2000) led to a number of modifications on the standard P. falciparum drug resistance assessment methods to allow a proper monitoring of emerging resistance to these new compounds. Different *in vitro*, *in vivo* and molecular methods were thus adapted to artemisinins efficacy studies (Witkowski and Amaratunga, 2015; Stepniewska et al., 2010a, Flegg et al., 2013, Ariey et al., 2014). Several point mutations on the *PfK13* propeller gene were found to be associated to the parasite clearance phenotype (Ariey et al., 2014).

Despite several studies on artemisinins efficacy in Africa, delay in parasite clearance time were rarely found (Borrmann et al., 2011, Ashley et al., 2014). Mutations on *PfK13* propeller were observed in very rare cases, in low frequency. In addition, the PfK13 mutations found in sub-Saharan Africa were mostly different from the ones associated with delay in parasite clearance time (PCT) in SE-Asia (Kamau et al., 2017; Maiga et al., 2012, Ouattara et al., 2015, Taylor et al., 2015, Ménard et al., 2016a). A few studies in eastern Africa found Asian mutations of PfK13 propeller resistance mutations but those mutations were not associated with prolonged parasite clearance (Borrmann et al., 2011, Tacoli et al., 2016).

Other studies either in Africa or even in Asia found delayed parasite clearance without *PfK13* propeller mutations (Muwanguzi et al., 2016; Neher, 2016 and MalariaGEN Plasmodium falciparum Community Project 2016; Mukherjee et al., 2017). Many factors related to both parasite genetic background and host immunity could explain differences observed in parasite clearance phenotypes between sub-Saharan Africa and south-east Asia (Djimde et al., 2003, Borrmann et al., 2011). In the contrary to the Asian parasites, little to no data were available for African field parasite sensitivity to ACT component drugs prior to their adoption for malaria treatment. Given the significant morbidity and mortality still associated with malaria in sub-Saharan Africa (Organization, 2018), efficient monitoring for efficacy of ACTs as well as their artemisinin's component is critical in Africa for malaria control and elimination strategies. This surveillance became even more critical for artesunate since it is now the first line therapy for the management of severe and complicated malaria cases in males and non-pregnant females. More sensitive tools may be needed to better characterize the phenotype of parasites and for early detection of *P. falciparum* resistance to artemisinin in Africa. Studies using qPCR to follow parasites clearance after ACTs found that, in addition to replicating parasite density derived from microscopy, this molecular method was able to detect submicroscopic parasitemia and give a clearer phenotype for parasite clearance time during field clinical trials in Africa (Beshir et al., 2010).

This present study compared the parasite clearance time after artesunate monotherapy treatment of uncomplicated malaria cases in two different areas of Mali, using both light microscopy and qPCR.

## 2 Materials and Methods

### 2.1 Study design and participants

Between October 2015 and March 2016, a prospective artesunate monotherapy study was conducted in Faladje and Bougoula-Hameau, two malaria endemic villages in Mali. Both villages have seasonal malaria transmission, are located in southern Mali but 400 kilometers apart.

Faladje is located at 80 kilometers West of Bamako (the capital city of Mali). Malaria transmission in Faladje is intense between July and October (Kayentao et al., 2009). Bougoula-Hameau is situated at 380 kilometers South of Bamako with rainy seasons lasting 6 months from May to October, and intense malaria transmission between July and November (Bouvier et al., 1997).

The trial enrolled patients of 6 months of age and older in both Faladje and Bougoula-Hameau.

The main inclusion criteria were an axillary temperature ≥ 37 °C or a reported history of fever in the previous 24 hours, a blood smear parasitemia between 2000 and 200,000 *P. falciparum* asexual forms per microliter, hemoglobin levels greater than 8.0 g/dl and no declared allergy to artemisinins. Volunteers with other acute illnesses or those with severe/complicated malaria were not included in this study. Participants were included after written informed consent, assent or parental consent for minors was obtained. A treatment course of 7 days of artesunate was administered at a first single dose of 4 mg/kg at inclusion day followed by a daily single dose of 2 mg/kg for the remaining of the treatment course. Patients were actively followed for 28 days according to modified standard protocols (Djimdé et al., 2008). Patients were hospitalized from inclusion to the resolution of malaria symptoms sustained by three consecutive negatives blood smears.

For the light microscopy clearance assessment, thick and thin blood smears were realized from finger pricks every 8 hours to also capture potential parasite burst from non-synchronic infections. This collection started from inclusion until three consecutively negative slides were obtained, as per the Worldwide Antimalarial Resistance Network (WWARN) adapted protocols (Flegg et al., 2013). After three consecutively negative slides, additional finger pricks were performed on days 7, 14, 21, 28 and/or on unscheduled days of clinical visit. Giemsa staining and microscopy were performed to measure asexual and sexual parasite densities. Parasitemia was assessed by counting the number of asexual forms per 200 leucocytes according to described protocols (WHO, 2013). Gametocytes density was estimated on 1000 leucocytes as described elsewhere (Von Seidlein et al., 2001). Patients were followed-up for 28 days with visits at days 7, 14, 21, 28 and any unscheduled days if the volunteer needed medical attention. Microscopy slide-reading was performed by two experienced readers, with discrepancies resolved by a third one.

Dried blood spots (DBS) were obtained at inclusion and every 8 hours until three consecutive negative slides were obtained, then at days 7, 14, 21, 28 and/or at unscheduled days of clinical visit.

PCR was performed to discriminate reinfections from recrudescent infections by using the molecular markers of parasite polymorphism *msp1, msp2* and *Ca1,* as described elsewhere (Snounou and Beck, 1998, Mugittu et al., 2006).

Treatment outcomes were assessed according to modified WHO protocol and classified as Early Clinical Failure (ECF), Late Clinical Failure (LCF), Late Parasitological Failure (LPF) and Adequate Clinical and Parasitological Response (ACPR) (WHO, 2013). Treatment failures were managed with ACTs or quinine intra-venously in 10% glucose followed by oral treatment as the patient status evolved. Cases that could not be handled in the local health center were referred to the nearest Hospital.

### 2.2 Parasite clearance time estimation

Light microscopy: the well-established parasite clearance time parameter was determined as the time from the first positive blood slide at inclusion to the time of the first negative slide followed by two consecutives negative slides. The WWARN parasite clearance estimator (PCE) was used to calculate parasite clearance slope half-life and the clearance rate constant (WWARN, 2010). Other parameters such as the proportion of patients having cleared 90% to 100% of their parasitemia by day 1 and the time to clear 50%, 90% or 100% of the day 0 parasitemia were also considered in this study.

qPCR: Samples were processed at the Malaria Research and Training Center in Bamako, Mali using a qPCR cycler (Light Cycler 480, Roche®, Mannheim, Germany). DNA was extracted from DBS collected prior to treatment, and at 24, 48 and 72 hours afterwards as previously described (Walsh et al., 1991). Parasitemia reduction was assessed by applying a relative quantification in the same sample of a parasite gene coding for the *Plasmodium* tRNA methionine (pgMET) and a human gene coding for the human β tubulin (HumTuBB). From the initial parasitemia, Parasite Reduction Rate (PRR) was calculated at different time point (24H, 48H and 72H) for each patient (Livak and Schmittgen, 2001, Beshir et al., 2010). Patients were considered as cleared when they reduced their initial parasitemia to a rate of 1/10,000, they were considered as having residual parasitemia when this rate was not met.

For each time point, a mean was calculated on patient's residual parasitemia per village.

### 2.3 Molecular markers of drug resistance

Several molecular markers for drug resistance were analyzed for their polymorphism in the two study sites. Day 0 dried blood spots collected on patients before drug administration were sent to whole genome sequencing at Wellcome Sanger Institute (Wellcome Genome Campus, Hinxton, UK). Polymorphisms for *Pfmdr1*, *Pf K13*, *Pfdhfr* and *Pfdhps* as well as *Pfmdr2*, *arps10*, and *ferredoxin* (parasite genetic background, PGB (Miotto et al., 2015)) *Pfcrt* K76 T genotype was analyzed by nested PCR as previously described (Djimde et al., 2001). A baseline prevalence for mutant parasites was determined for selected SNPs on those different genes for each study site.

### 2.4 Data analysis

Data was included in the final analysis when the patient received the full dose of treatment, had not received another antimalarial treatment and attended all follow up visits. The sample size was calculated based on the in vivo clinical and parasitological failure rate around 17% on Day 28 using artemether-lumefantrine as the reference drugs in Mali (Sagara et al., 2016). Assuming a random error estimate at 5%, the α-level of test at 5% and the power (1-β) of 80%, the number of evaluable volunteers necessary for the study was 216 for both sites.

Proportions, medians and means were measured by descriptive statistics and compared by the χ^2^ test, the student's *t* test or the Wilcoxon-Mann Whitney test as appropriate. The main parameters affecting parasite clearance such as patient age and the initial parasitemia were included in the model as confounding factors as well as sex, hemoglobin levels, gametocyte density and fever at enrolment. Data were entered in excel 2011 and STATA version 11.2 for statistical analysis. The genotyping data for each molecular marker was extracted from the MalariaGEN genetic report card based for these locations. Missing data were removed and the frequency of variations in each molecular marker was calculated using the dplyr package implemented in R [Hadley Wickham, Romain François, Lionel Henry and Kirill Müller (2019). R package version 0.8.3.]

The study protocol was reviewed and approved by the Ethics Committee of the Faculty of Pharmacy and the Faculty of Medicine and odonto-stomatology, University of Sciences Technics, Technologies of Bamako, Mali.

## 3 Results

### 3.1 Clinical/parasitological outcomes

**Fig. 1:**
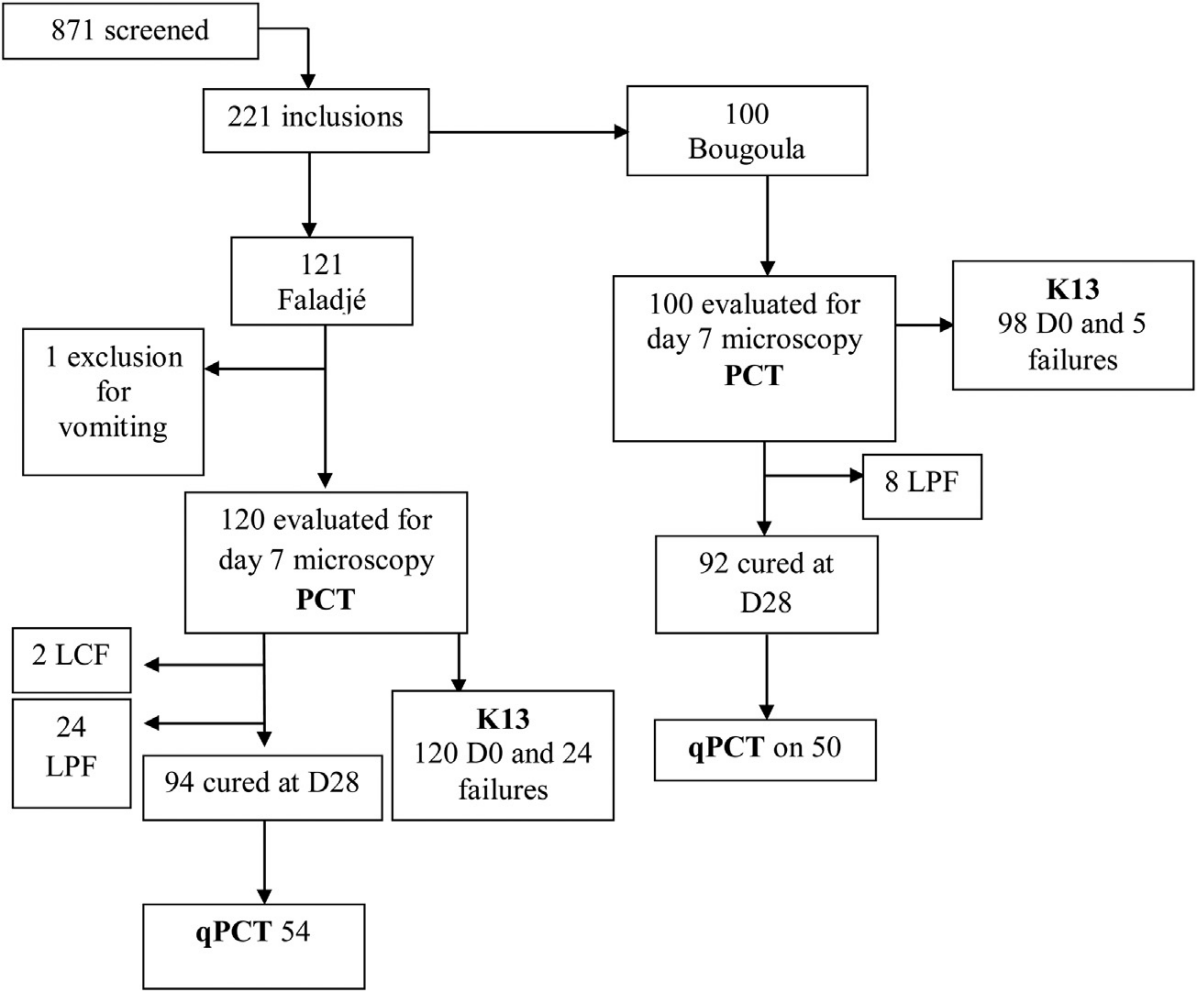
Study profile.

Overall 221 volunteers were included in this study, 121 from Faladje and 100 from Bougoula-Hameau. One exclusion case for persistent vomiting after drug administration was observed in Faladje. No other losses to follow up happened for the two study sites (Fig. 1). At enrolment, the two study populations were comparable for sex ratio, median asexual parasitemia, mean hemoglobin, proportions of anemia, fever and gametocyte carriage as well as mean gametocyte density (Table 1).

**Table 1:** Demographic characteristics at enrolment

**Parameters**	**Faladje**	**Bougoula-Hameau**	***p*****value**
Age, median in year (IQR)	8.5 (5.5-11.2)	9 (6.8-11.7)	0.2
Male sexe n (%)	55 (45.8%)	53 (53%)	0.4
Pf * parasitemia/ul, median (IQR)	25,680 [14,540-53,720]	29,140 [19,080-53,820]	0.5
Pf* Gametocyte/ul, mean (SD)	40 [24]	71 [60.8]	0.4
Pf* Gametocyte carriers, n (%)	3 (2.5%)	8 (8%)	0.06
Hemoglobin, mean (SD)	11.01 [1.6]	11.1 [1.5]	1
Anemia, n (%)	60 (50%)	44 (44%)	0.5
Fever, n (%)	57 (47.5%)	56 (56%)	0.3

* : Plasmodium falciparum

There were 2 LCF and 24 LPF cases (78.3% ACPR) registered for Faladje versus 8 LPF (92% ACPR) for Bougoula-Hameau (p = 0.01). After PCR correction, all recurrent infections were new infections, bringing the artesunate PCR corrected cure rate to 100% in both villages.

### 3.2 Parasite clearance assessed by light microscopy

**Fig. 2:**
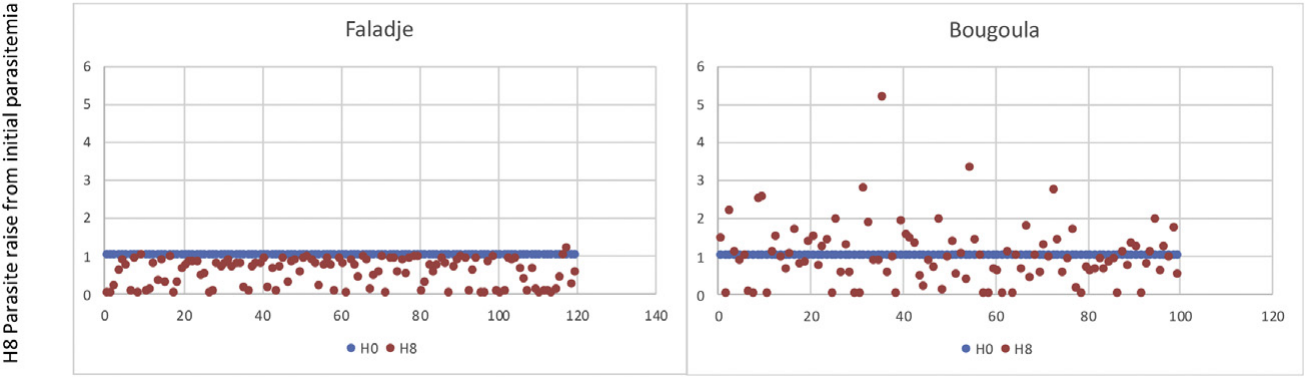
Distribution of H8 parasitemia around the initial H0 parasitemia value of 1.

At eight hours after treatment initiation, only 1.7% of Faladje patients had an increase in the baseline parasitemia while in Bougoula-Hameau 39% of patients had an increase in the baseline parasitemia (p < 0.0001) (Fig. 2). However, this was followed in all cases by a rapid decrease of the general parasite density between 16 to 24 hours after treatment initiation. At 24 hours following artesunate treatment, 97.5% of Faladje patients had detectable parasitemia against 72% in Bougoula-Hameau (P < 0.0001). At 72 hours post-treatment, patients from neither study sites had a microscopically detectable parasitemia. The median times to clear 90% of the initial parasitemia was 11.8 hours in Faladje vs 10.6 hours for Bougoula-Hameau (P = 0.04). The median time to clear all parasitemia was significantly longer in Faladje with 40 hours compared to Bougoula-Hameau with 32 hours (P < 0.0001) (Table 2).

**Table 2:** Parasite clearance parameters using light microscopy

	**Faladje**	**Bougoula-Hameau**	***p*****value**
Proportion of patients with cleared parasitemia 24 h after treatment	3 (2,5%)	28 (28%)	<0.0001
Time in hour to clear 50% of the parasitemia, median (IQR)	6,7 [4,6-10,6]	4,6 [3,7-5,5]	<0.0001
Time in hour to clear 90% of the parasitemia, median (IQR)	11,8 [8,9-15]	10,6 [9-12,2]	0.04
Total clearance time, median (in hour)	40	32	P < 0,0001

**Fig. 3:**
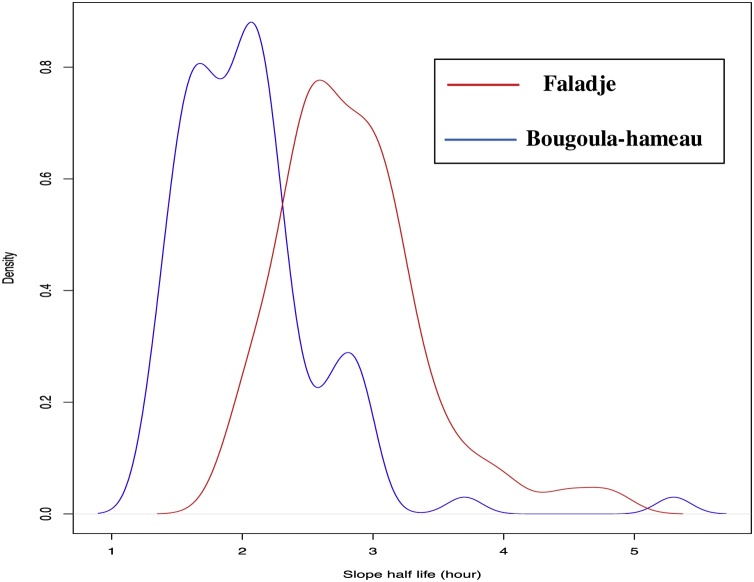
*P. falciparum* clearance half-life distribution post-artesunate monotherapy in Faladje and Bougoula-hameau, Mali.

Using the WWARN PCE tool, the parasite clearance slope half-life was significantly longer in Faladje with a median of 2.8 hours than in Bougoula-Hameau with 2 hours (P < 0.0001) (Fig. 3). There was a correlation between parasite clearance and both age (P = 0.02) and initial parasitemia (P = 0.03) in Bougoula-Hameau. This was not observed in Faladje.

### 3.3 Parasite clearance assessed by qPCR

Complete qPCR results were obtained for 104 (54 for Faladje and 50 for Bougoula-Hameau) out of 111 analyzed samples.

**Fig. 4:**
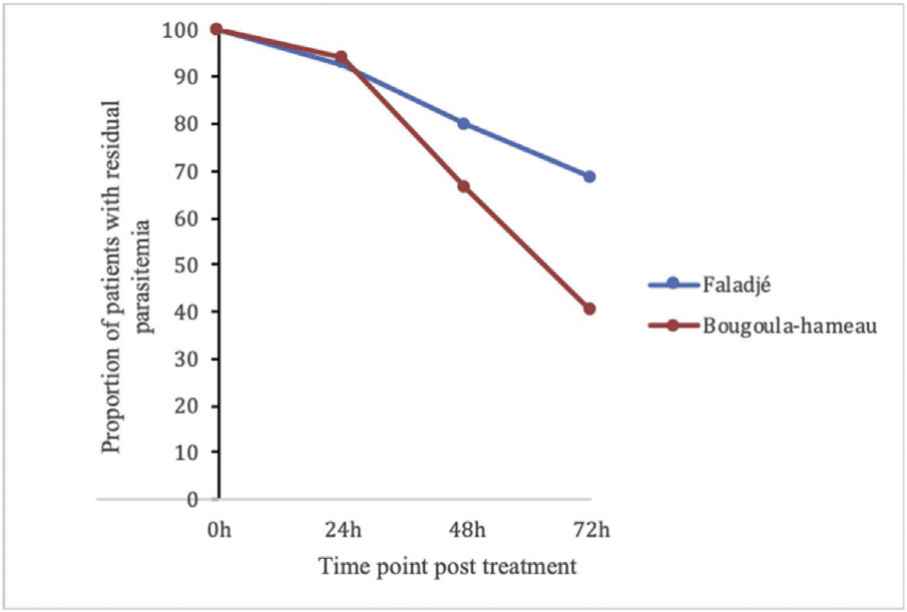
Proportion of patient with residual parasitemia measured by qPCR in Faladje and Bougoula-hameau. At 24 hours and 48 hours the two villages were comparable with, Faladje presenting 92,6% and Bougoula-hameau 94% (p = 0.7) at 24 hours and at 48 hours Faladje had 79,6% and Bougoula-hameau 66% (p = 0.1). Proportions at 72 hours were different with 68.5% in Faladje and 40% in Bougoula-hameau (p = 0.003) *

For the first 24 and 48 hours following artesunate treatment, the proportions of patients with residual parasitemia was comparable for the two study sites (Fig. 4). At 72 hours the proportion of patients with residual parasitemia was 68.5% in Faladje and 40% in Bougoula-Hameau (P = 0.003). The mean residual parasitemia of all patients at 72 hours was 2.9 and 0.08 for Faladje and Bougoula-Hameau, respectively (P = 0.001)

### 3.4 Molecular markers of drug resistance

Only one non-synonymous *PfK13* mutation was found in Bougoula-Hameau (A578S) (N = 98), while no such mutation was found in Faladje (N = 118). The prevalence of *Pfcrt* K76 T, *Pfmdr1* N86Y, *Pfdhfr-Pfdhps* quadruple mutations in Faladje and Bougoula-Hameau were comparable (Table 3). One *Pfdhfr-Pfdhps* quintuple mutant was observed in Bougoula-Hameau only. There was no difference in the prevalence of PGB haplotypes between the two sites (Table 3).

**Table 3:** Molecular markers of drug resistance in the two study sites

				**Faladje**	**Bougoula-Hameau**	
**Genes**	**Antimalarial**	**Amino Acid Positions**	**Wild Type Haplotype**	**Mutants %(N)**		p
PfK13	artemisinin	any mutation seen in BTB/POZ		0 (120)	1 (100)	0.2
Pfdhfr-Triple-mutation	pyrimethamine	51,59,108	NCS	83.7(140)	79.6(166)	0.3614
Pfdhfr-Pfdhps quadruple mutation	Sulfadoxine-pyrimethamine	51,59,108, 480	NCS-A	52.6(140)	52(166)	0.9
Pfdhfr-Pfdhps quintuple mutation	Sulfadoxine-pyrimethamine	51,59,108, 480, 540	NCS-AK	0 (140)	0.6 (166)	0.3
Pfmdr1	chloroquine, amodiaquine, lumefantrine, mefloquine	86	N	40(118)	38.8(163)	0.8
Pfcrt	chloroquine	76	K	30(110)	23.7(71)	0.3
PGB (ART-R genetic background), arps10, ferredoxin, Pfcrt, Pfmdr2	artemisinin	arps10-127; ferredoxin-193; Pfcrt-326, 356; Pfmdr2-484	VDNIT	32.6(126)	29.6(151)	0.5

## 4 Discussion

Following artesunate monotherapy for uncomplicated falciparum malaria, parasite clearance time (PCT) was significantly longer in Faladje than in Bougoula-Hameau, using both light microscopy and qPCR. Although, the nearly 3-hour slope half-life of parasitemia clearance of Faladje did not reach the current 5-hour threshold for artemisinin resistance described in SEA (Dondorp et al., 2009), this observation merits careful consideration. The PCT found in Bougoula-Hameau is similar to PCTs reported ten years earlier in the same site (Maiga et al., 2012) as well as in Kenieroba, another village of Mali (Lopera-Mesa et al., 2013). It is also comparable to the one observed in a similar study conducted in DR Congo (Ashley et al., 2014). As with light microscopy qPCR showed significantly slower PCT in Faladje than in Bougoula-Hameau. Furthermore, there were significantly more patients with qPCR detectable parasitemia 3 days after treatment initiation in Faladje than Bougoula-Hameau. Residual parasitemia detected by qPCR was described as being associated with malaria transmission and with the development of drug resistance (Beshir et al., 2013). The rate of 72 hour - parasitological failure by qPCR varied between 40% and 68% while the rate of 72 hour - parasitological failure by microscopy was 0% in both villages, indicating that qPCR would be a better tool for the early detection of artemisinin resistance in these settings.

A number of hosts, parasite and environmental factors might affect parasite elimination from the bloodstream. Differences in malaria transmission levels could impact the PCT (Stepniewska et al., 2010b). In our two study sites malaria transmission was shown to be comparable (Bouvier et al., 1997; Kayentao et al., 2009). Therefore, the difference in PCT between these two villages are probably not due to differences in transmission. Because both Faladje and Bougoula-Hameau are regularly used as antimalarial drug testing sites, this could have an impact on parasites susceptibility. However, the sites are being used at similar frequency for these studies and solely by our research team.

Host immunity is known to significantly contribute to parasite clearance (Djimde et al., 2003, Ataide et al., 2017). Age was suggested to be correlated with host immunity (Djimde et al., 2003, Lopera-Mesa et al., 2013). Because the available data suggest that transmission and age distributions are similar between the two sites, it is reasonable to expect similar overall levels of human host immunity in the two villages. Furthermore, a recent study indicated that prevalence of anti *P. falciparum* MSP1-42 and *P. falciparum* AMA-1 antibodies were similar between the two areas where the respective villages are located (Rogier et al., 2017). More studies will be required to clarify the potential role of host immunity in the observed differences in PCT. Indeed the 5-hour slope half-life threshold that was shown to correlate with artemisinin decreased efficacy in South-East Asia (Ashley et al., 2014) may not hold in sub-Saharan Africa that is still presenting with the highest transmission intensity (World Health Organization, 2018).

Ethnic differences have been shown to affect susceptibility to malaria. Indeed, Fulani are less susceptible to malaria than sympatric Mossi and Dogon ethnic groups in Burkina Faso and Mali, respectively (Modiano et al., 1999, Dolo et al., 2012). However, more than 95% of residents of Faladje and Bougoula-Hameau share the same last name, which indicates that they are most likely of similar ethnic background (our unpublished observations).

Only *PfK13* A578S mutation was found in one infection in our dataset. This mutation was observed in previous studies in Mali (Ouattara et al., 2015) and in other African countries (Kamau et al.; Ashley et al., 2014, Ménard et al., 2016b). However, *PfK13* A578S appears not to be involved with artemisinin resistance (Kamau et al.; Muwanguzi et al., 2016). We note that an increasing number of studies describe artemisinins resistance in the absence of *PfK13* mutations (Madamet et al., 2017, Ocan et al., 2018). This highlights the possibility for Plasmodium parasites to evolve other resistance mechanisms independently or in addition to *PfK13* gene (Kheang et al., 2017, Mukherjee et al., 2017).

A longer PCT could have been attributable to parasite genetic background of resistance (Miotto et al., 2015). Indeed the village of Faladje had 45% of *in vivo* chloroquine resistance, the highest rate ever documented in Mali (Sangho et al., 2004) wile in the same period the *in vivo* resistance in Bougoula-Hameau was 17.9% (our unpublished data). Nevertheless, prevalence of current molecular markers of antimalarial drug resistance was comparable for the two villages (Table 3). In addition, the genetic background associated to artemisinin resistance is similar for both sites.

Therefore, the known genetic background of drug resistance does not explain the PCT differences observed in Faladje and Bougoula-Hameau.

Despite these differences observed in clearance time in the two study areas, artesunate monotherapy showed 100% corrected ACPR and no patients had detectable parasitemia by light microscopy 72 hours after treatment, indicating high clinical efficacy of this drug in both villages during this study period.

While less than 2% of patients had a rise in parasitemia eight hours after treatment in Faladje, two out of every five patients had a rise in parasitemia at the same time point. This initial rise of parasitemia shortly after artemisinin treatment, which could be related to the presence of non-synchronous parasite populations in patients had been described (Silachamroon et al., 2001, Maiga et al., 2012). The increased parasitemia observed at 8H after treatment might be due to new merozoites from newly ruptured schizonts (Saralamba et al., 2011). The phenomenon could also reflect multiplicity of infection with different clones being at different developmental stages. Other parasite factors such as parasitemia or host factors such as immunity could also be involved in lack of synchronicity of *P.falciparum* infection in people (White, 2017). The mechanisms involved in the observed sharp difference between the two villages in terms of rise of initial parasitemia after artesunate treatment requires further investigations.

Slower parasitemia clearance after artesunate monotherapy in a village known for higher chloroquine-resistance could be early signs of diminished response to artemisinins in Mali. Bearing in mind the facts that the 5-hour slope half-life threshold for artemisinin resistance/tolerance was initially established in south-East Asia and that immunity and other host factors may mask increases in PCT in higher malaria transmission settings, more sensitive tools such as qPCR may be better indicated for the monitoring of the emergence of artemisinin resistance in sub-Saharan Africa.

## Financial support

Field studies were supported by a Wellcome Sanger International Fellow to AAD. AAD is currently supported through the DELTAS Africa Initiative, (DELGEME grant 107740/Z/15/Z). Malaria Research and Capacity Development (MARCAD), a DELTAS Program [DEL-15-010] supported Aminatou Kone through a Post-doctoral fellowship. The Developing Excellence in Leadership and Genetics Training for Malaria Elimination in sub-Saharan Africa (DELGEME) a DELTAS Program [DEL-15-002] supported Aoua Coulibaly for a PhD fellowship. The DELTAS Africa Initiative is an independent funding scheme of the African Academy of Sciences (AAS)’s Alliance for Accelerating Excellence in Science in Africa (AESA) and supported by the New Partnership for Africa's Development Planning and Coordinating Agency (NEPAD Agency) with funding from the Wellcome Trust [grant 107741/A/15/Z] and the UK government. The views expressed in this publication are those of the author(s) and not necessarily those of AAS, NEPAD Agency, Wellcome Trust or the UK government.

A.K received support from the World Academia of Sciences (TWAS) for providing financial support through the grant number Ref.: 17-346 RG/BIO/AF/AC_I–FR3240297741.

Corresponding author: Professor Abdoulaye A. Djimde, adjimde@icermali.org, Malaria Research and Training Center, Faculty of Pharmacy, University of Science, Techniques and Technology, Bamako, Mali

## Declaration of Conflict of interest

Pr. Djimde has nothing to disclose.
